# Fructose, but not glucose, impairs insulin signaling in the three major insulin-sensitive tissues

**DOI:** 10.1038/srep26149

**Published:** 2016-05-19

**Authors:** Miguel Baena, Gemma Sangüesa, Alberto Dávalos, María-Jesús Latasa, Aleix Sala-Vila, Rosa María Sánchez, Núria Roglans, Juan Carlos Laguna, Marta Alegret

**Affiliations:** 1 Department of Pharmacology and Therapeutic Chemistry, School of Pharmacy, University of Barcelona, Spain; 2 Institute of Biomedicine, University of Barcelona, Spain; 3 IMDEA Food. CEI UAM+CSIC, Madrid, Spain; 4 CIBER Fisiología de la Obesidad y Nutrición (CIBEROBN), Instituto de Salud Carlos III (ISCIII), Spain; 5 Lipid Clinic, Endocrinology and Nutrition Service, Hospital Clínic, Institut d’Investigacions Biomèdiques August Pi i Sunyer (IDIBAPS), Barcelona, Spain

## Abstract

Human studies support the relationship between high intake of fructose-sweetened beverages and type 2 diabetes, but there is a debate on whether this effect is fructose-specific or it is merely associated to an excessive caloric intake. Here we investigate the effects of 2 months’ supplementation to female rats of equicaloric 10% w/v fructose or glucose solutions on insulin sensitivity in target tissues. Fructose supplementation caused hepatic deposition of triglycerides and changed the fatty acid profile of this fraction, with an increase in monounsaturated and a decrease in polyunsaturated species, but did not cause inflammation and oxidative stress. Fructose but not glucose-supplemented rats displayed an abnormal glucose tolerance test, and did not show increased phosphorylation of V-akt murine thymoma viral oncogene homolog-2 (Akt) in white adipose tissue and liver after insulin administration. In skeletal muscle, phosphorylation of Akt and of Akt substrate of 160 kDA (AS160) was not impaired but the expression of the glucose transporter type 4 (GLUT4) in the plasma membrane was reduced only in fructose-fed rats. In conclusion, fructose but not glucose supplementation causes fatty liver without inflammation and oxidative stress and impairs insulin signaling in the three major insulin-responsive tissues independently from the increase in energy intake.

The incidence of type 2 diabetes and insulin resistance is increasing worldwide, a trend that is largely attributable to lifestyle choices^1^. Unhealthy nutritional patterns such as high consumption of simple carbohydrates are related to these metabolic disorders. The use of fructose as a sweetener in beverages and processed foods (either as high fructose corn syrup or sucrose) contributes to an excessive dietary carbohydrate consumption. Studies in humans show that top consumers ingest over 100 g/day of fructose as added sweetener, which based on a 2000 kcal/day diet means that they derive more than 20% of their calories from fructose^2^. Due to its particular metabolic fate, fructose exerts specific effects on lipid and carbohydrate metabolism^3^,^4^; moreover, fructose in liquid form may be more harmful than in solid form, as the excess energy is not fully compensated by a decrease in food intake^5^. Several studies in humans support the relationship between high intake of fructose and hyperglycaemia, insulin resistance and type 2 diabetes^3^,^6^,^7^. However, it has been suggested that fructose is no worse than glucose in causing adverse cardiometabolic effects, even under equicaloric conditions^8^, and that the metabolic alterations observed should be attributed to the excess of energy intake and to changes in body weight^9^.

The rat is a good model to study the effects of fructose on carbohydrate metabolism in humans^4^. Unlike other species, rats and humans lack the intestinal enzyme that converts a substantial part of ingested fructose into glucose, and the administration of fructose-enriched diets to rats induces metabolic alterations that closely resemble the human metabolic syndrome. Thus, although caution is needed when extending results of animal studies to humans, rats are useful models to understand the molecular mechanisms underlying the metabolic derangements caused by high-fructose diets. We have previously shown that the administration of a 10% w/v fructose solution to female rats caused hyperinsulinaemia, glucose intolerance, reduced insulin receptor substrate-2 (IRS-2) hepatic expression, and hepatic steatosis, which we attributed to the induction of carbohydrate response element binding protein (ChREBP)^10^,^11^. In these studies, fructose was administered for 14 days, which is a short treatment period given that fructose consumption patterns in humans extend over many years.

Therefore, we studied the effects on insulin signaling in target tissues of a 2-month period of fructose supplementation, comparable to 6 human years of daily fructose consumption^12^. We investigated whether there is a dissociation between fatty liver and insulin resistance and inflammation, as reported in mice with ChREBP overexpression^13^. In addition, to distinguish between effects related to increased caloric intake and the specific effects of fructose, we performed a second set of experiments including a group supplemented with liquid glucose under equicaloric conditions.

## Methods

### Animals and diets

Female Sprague-Dawley rats obtained from Charles River (Barcelona, Spain) were used for all experiments. Rats were maintained under conditions of constant humidity (40–60%) and temperature (20–24°) with a light/dark cycle of 12 hours. Procedures were conducted following the ‘Principles of laboratory animal care’ (NIH publication no. 85–23, revised 1985) and in accordance with the guidelines established by the University of Barcelona’s Bioethics Committee (Autonomous Government of Catalonia Act 5/1995, July 21). All the experimental procedures involving animals were approved by the Ethical Committee of Animal Experimentation of the University of Barcelona (approval n° 5448).

#### Experiment 1

20 rats aged 8 weeks were randomly assigned either to a control group (no supplementary sugar, n = 8) or a fructose-supplemented (10% w/v in drinking water) group (n = 12) for 2 months^14^. At the end of intervention, after a 2-h fast, the animals were anaesthetised with isoflurane and blood taken from the hepatic portal vein was collected in endotoxin-free tubes. Then, rats were euthanized by exanguination and liver samples were collected and stored as described^15^. The gastrocnemius muscle of both legs, and visceral white adipose tissue (vWAT, including retroperitoneal, perirenal and perigonadal adipose tissue) was collected, immediately frozen in liquid nitrogen, and stored at −80 °C until needed.

#### Experiment 2

Another cohort of rats was used to perform a glucose tolerance test (GTT) and to examine their response to the administration of a bolus of insulin. Study duration was the same as above, but the experimental design also included a group supplemented with a glucose solution (n = 10 animals/group). Liquid and food intake were measured every day, caloric consumption was calculated, and the concentration of the glucose solution to be prepared for the next day was calculated in order to match the amount of calories ingested by the fructose group.

The GTT was performed as described^10^, with some modifications. Briefly, after a 6-h fast, the rats were anesthetized, and following the collection of an unchallenged sample (time 0), a glucose solution of 2 g/kg body weight was administered into the peritoneal cavity. During the test, blood was collected from the tail vein at 15, 30, 60, 90 and 120 min after glucose administration. Glucose measurements were performed using a hand-held glucometer. Plasma insulin levels were measured at baseline, 15 and 120 min post glucose administration by using a rat insulin ELISA kit (Millipore, Billerica, MA). The area under the curve (AUC) values for glucose and insulin concentrations were calculated by the trapezoidal method, using 0 as the baseline.

One week after the GTT 12-h fasted animals were anaesthetised with ketamine:xylazine (90:10 mg/kg body weight) and intraperitoneally injected with 0.15 units of insulin/g body weight (Humulina® Regular, Lilly, Madrid, Spain)^16^. 15 minutes later, blood was obtained from the hepatic portal vein, and samples from liver, gastrocnemius muscle and vWAT were collected as described for experiment 1.

### Blood/plasma lipids and insulin analysis

Blood triglycerides were measured using an Accutrend® Plus System glucometer (Cobas, Roche Farma, Barcelona, Spain). Plasma cholesterol was determined by Cholesterol CHOD-POD colorimetric test N° 1001091 (Spinreact, Girona, Spain). Non-esterified fatty acids (NEFA) were determined using a plate-based colorimetric enzymatic assay (5620-01) from Bioo Scientific (Austin, TX, USA). Concentrations of insulin in plasma were determined using an enzyme-linked immunosorbent assay kit (Millipore, Billerica, MA).

### Quantitative determination of alanine aminotransferase (ALT)

To determine ALT activity in plasma samples we used the ALT/GPT Spinreact kit from Spinreact (Girona, Spain), based on the determination of the rate of decrease in NADH concentration, measured photometrically.

### Lipidomic analysis of liver triglyceride fatty acid composition

Liver lipids were extracted and analysed as described previously^17^. For fatty acid determination, internal standard of C15:0 triglyceride was added to samples of liver homogenates followed by extraction of the total lipid material with chloroform/methanol (2:1 v/v). Triglycerides were isolated by solid-phase extraction as described in Burdge *et al*.^18^. Fatty acid methyl esters were prepared by incubation with acidified methanol and were separated by gas chromatography with an Agilent 7890 Gas Chromatograph HP 6890 equipped with a 30 m × 0.25 μm × 0.25 mm SupraWAX-280 capillary column (Teknokroma, Barcelona, Spain), an autosampler, and flame ionisation detection. Stearoyl-CoA desacturase (SCD1) activity was calculated as the ratio of palmitoleic to palmitic acid concentrations in hepatic triglycerides (C16:1 n-7/C16:0), as this index reflects hepatic SCD1 expression more closely than the ratio oleic to stearic acid^19^.

### Endotoxin assay

Blood samples from the hepatic portal vein collected in endotoxin-free tubes were heated at 70 °C for 5 minutes. Endotoxin plasma levels were measured using a limulus amoebocyte lysate assay with a concentration range of 0.015–1.2 EU/mL (Charles River, L’Arbresle, France).

### Histological studies

Necrosis and fibrosis were analyzed in liver sections stained with haematoxylin-eosin and trichromic acid, respectively. Images, acquired with an Olympus BX43 microscope, were interpreted at BioBanc (Banc de tumors-IDIBAPS, Barcelona Spain).

### RNA preparation and analysis

Total RNA was isolated by using the TrizolR reagent (Invitrogen, Carlsbad, CA, USA), in accordance with the manufacturer’s instructions. RNA concentration and purity were measured spectrophotometrically using the NanoDrop® ND-1000 Spectrophotometer (Thermo Scientific). The ratio of absorbances at 260/230 and at 260/280 were used as indicators for RNA purity. RNA integrity was determined by running samples on 1% agarose gels stained with ethidium bromide. RNA quality was checked by the presence of sharp 28S and 18S rRNA bands at a ratio 2:1. Specific mRNAs were assessed by real-time reverse transcription polymerase chain reaction (RT-PCR) using SYBR green PCR Master Mix, specific primers and the Applied Biosystems One-Step Plus sequence detection system (Applied Biosystems, Foster City, CA, USA). As internal control, TATA box binding protein (tbp) was used. Primer sequences and PCR product length are listed in Supplementary Table 1.

### Analysis of microRNA

Total RNA, including small RNAs, was isolated using the miRNeasy minikit (Qiagen). For microRNA (miRNA) expression quantification, miRNAs were reverse-transcribed using the miScript II reverse transcription kit (Qiagen). Specific primers were used for each miRNA (Qiagen), and real-time PCR was performed using the miScript SYBR Green PCR kit (Qiagen) on a 7900 HT Fast Real-Time PCR System (Applied Biosystems). Relative expression was calculated by the 2^−ΔΔCt^ method using RNU6 for normalisation. Only miRNAs validated for the target genes were analysed. Selection of validated miRNAs was assessed using the MirWalk 3^20^ and miRTarbase 4^21^ databases.

### Preparation of protein extracts

Total and nuclear protein extracts from liver and muscle were obtained by the Helenius method^22^. Adipose tissue samples were micronized through freezing with liquid nitrogen and grinding with a mortar. For total protein extraction, lysis buffer with proteases, phosphatases and acetylase inhibitors (50 mM Tris–HCl pH = 8, 150 mM NaCl, 1% Igepal, 10 mM NaF, 1 mM EDTA, 1 mM EGTA, 2 mM Nappi, 1 mM PMSF, 2 μg/mL leupeptin, 2 μg/mL aprotinin, 1 mM Na3VO4, 10 mM NaM, 1 μM TSA) were added to micronized tissue and homogenized for 1.5 h at 4 °C. Then, samples were centrifuged at 15,000 × g for 15 min at 4 °C and supernatant was collected. Protein concentrations were determined by the Bradford method^23^. To detect GLUT4 protein translocation to the plasma membrane, a subcellular fraction enriched with plasma membrane proteins was prepared from rat muscle samples by sequential centrifugation^24^.

### Western blot analysis

30 μg of different protein fractions from rat tissues was subjected to SDS-polyacrylamide gel electrophoresis. Proteins were then transferred to Immobilon polyvinylidene difluoride transfer membranes (Millipore, Billerica, MA, USA), blocked for 1 h at room temperature with 5% non-fat milk solution in 0.1% Tween-20-Tris-buffered saline (TBS), and incubated as described previously^15^. Detection was performed using the ECL chemiluminescence kit for HRP (Amersham GE Healthcare Europe GmbH, Barcelona, Spain). To confirm the uniformity of protein loading, blots were incubated with β-tubulin or β-actin antibodies (Sigma-Aldrich, St. Louis, MO, USA) as a control. Primary antibodies for phospho-and total AKT, phospho- and total AS160, phospho-ERK, GLUT4, phospho-PKA and phospho-TSC-2 were supplied by Cell Signaling (Danvers, MA, USA), those for 14-3-3, ChREBP, phospho-and total FoxO1, IRS-1, IRS-2, MKP-3 and phospho-SGK-1 were obtained from Santa Cruz Biotechnologies (Dallas, TX, USA), and antibody against phospho-mTOR was from Millipore (Billerica, MA, USA).

### Statistical analysis

The results are expressed as the mean of n values ± standard deviation. Plasma samples were assayed in duplicate. Gaussian distribution of the data was verified using the Kolmogorov-Smirnov normality test, and significant differences were established by two-tailed unpaired t-test, ANOVA test, or a non-parametric test, as appropriate (GraphPad Prism Software V5). The level of statistical significance was set at p ≤ 0.05.

## Results

### Fructose induces hepatic steatosis and modifies liver lipid composition through ChREBP induction

**Figure 1:**
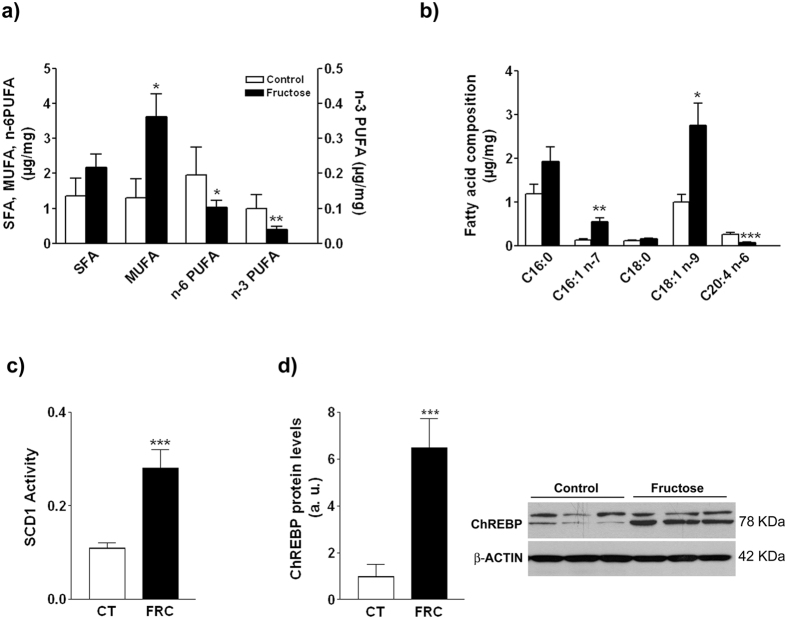
Triglyceride fatty acid content (chemical groups (**a**), selected species (**b**), and SCD1 activity index estimated by the ratio 16:1/16:0 (**c**), and western-blot analysis of ChREBP in nuclear extracts (**d**) in hepatic samples from control and fructose-supplemented rats from experiment 1. Bars represent the mean ± sd of values obtained from n = 6 (control) and n = 8 (fructose-fed) animals. A representative western blot corresponding to 3 different control and fructose-fed rats is shown.*p < 0.05; **p < 0.01; ***p < 0.001 (unpaired t test).

Initially we used liver samples from a previous study^14^ to assess the effects of 2 months’ 10% (w/v) fructose supplementation on the fatty acid profile of hepatic triglycerides. Fructose supplementation (experiment 1) did not modify plasma cholesterol concentration, but caused hypertriglyceridaemia and hepatic steatosis (Table 1, previous study^14^). Total monounsaturated fatty acids (MUFA) in the hepatic triglyceride fraction were significantly increased (x2.8-fold, p < 0.05, Fig. 1a), as a result of significant increases in both palmitoleic acid (C16:1 n-7) (x4.1-fold, p < 0.01) and oleic acid (C18:1 n-9) (x2.7-fold, p < 0.05) (Fig. 1b). Consistently, SCD1 activity estimated by the ratio of product to substrate (C16:1 n-7/C16:0) was increased x2.5-fold (p < 0.001) (Fig. 1c). In contrast, both n-3 and n-6 polyunsaturated fatty acids (PUFA) were decreased x0.4 and x0.5-fold, respectively (p < 0.05, both) (Fig. 1a). The latter was mostly explained by a x0.3-fold reduction (p < 0.001) in arachidonic acid (C20:4 n-6) (Fig. 1b). The amount of ChREBP in nuclear extracts was increased x6.5-fold (p < 0.001) compared with control rats (Fig. 1d).

### Fructose reduces hepatic expression of IRS-2

**Figure 2:**
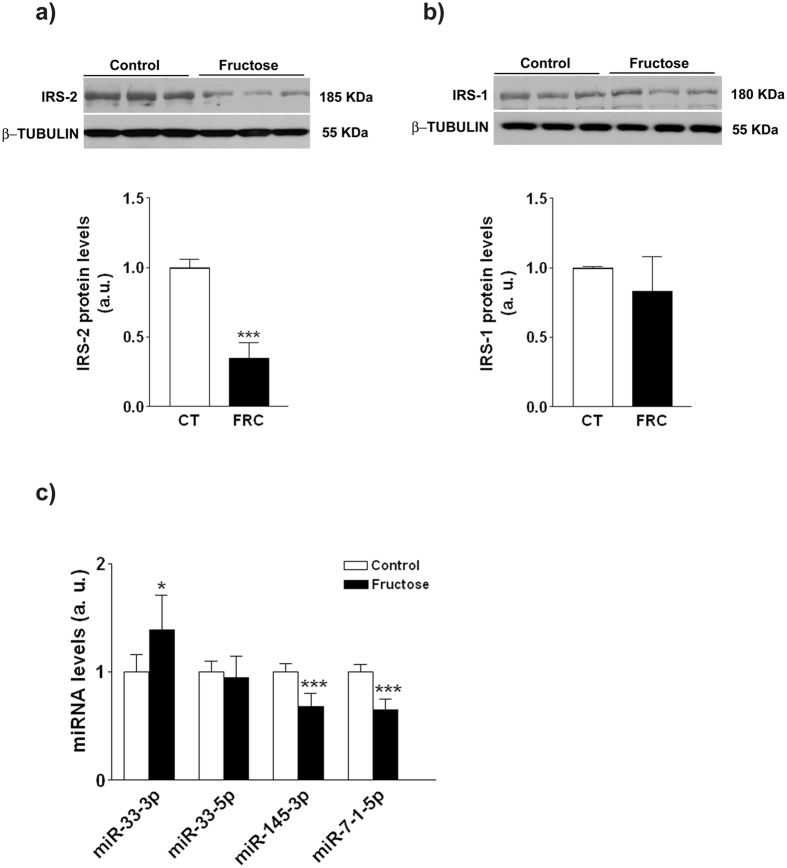
Expression of proteins and microRNAs involved in hepatic insulin signaling. IRS-2 (**a**) and IRS-1(**b**) protein levels determined by western blot. (**c**) RT-PCR analysis of miR-33-3p, miR-33-5p, miR-145-3p and miR-7-1-5p. Bars represent the mean ± sd of values obtained from n = 6 (control) and n = 8 (fructose-fed) animals from experiment 1. Representative western blots corresponding to 3 different control and fructose-fed rats are shown.*p < 0.05; ***p < 0.001 (unpaired t test).

Fructose supplementation led to a drastic decrease in the amount of hepatic IRS-2 (x0.35-fold, p < 0.001) which was not accompanied by a compensatory increase in IRS-1 (Fig. 2a,b). To gain an insight into the mechanisms by which fructose administration leads to reduced IRS-2, we analysed some miRNAs which control the expression of proteins involved in insulin signaling. As shown in Fig. 2c, miR-33-3p expression was increased (x1.4-fold, p < 0.05), whereas the expression of miR-145-3p and 7-1-5p was reduced (x0.65-0.68-fold, p < 0.0001).

### Hepatic steatosis in fructose-supplemented rats is dissociated from inflammation and oxidative stress in liver and vWAT

The livers of fructose-supplemented rats showed a reduced mRNA level (x0.45-fold, p = 0.05) of nuclear factor-E2-related factor-2 (*nrf2*), a cellular sensor for oxidative stress (Table 2). Expression of the antioxidant genes glutathione peroxidase 1 (*gpx1*) and superoxide dismutase 2 (*sod2*) was not significantly altered (Table 2).

**Figure 3:**
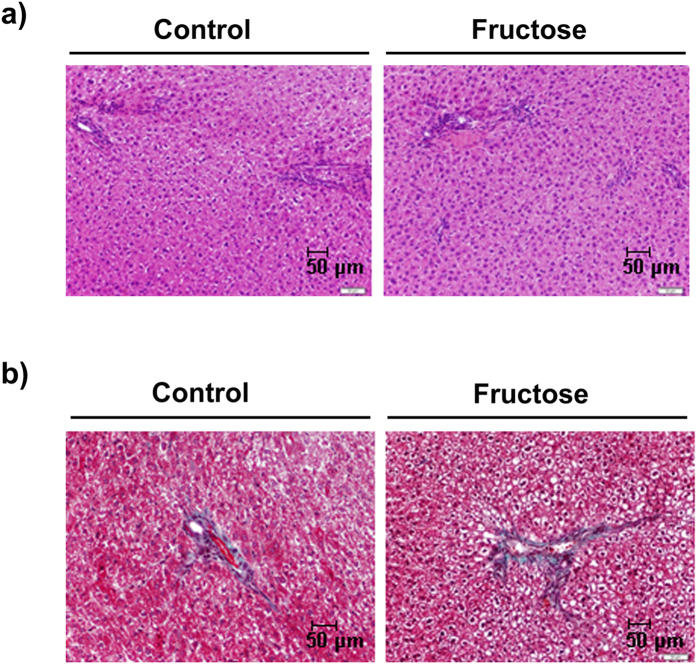
Histological study of liver sections from control and fructose-supplemented rats stained with H&E (**a**) and Masson’s trichrome (**b**). Images are representative from n = 4/group, experiment 1 (magnification 20X).

Endotoxin levels in portal vein blood samples, and hepatic mRNA expression of toll-like receptor-4 (*tlr4*) and its downstream myeloid differentiation factor 88 (*myd88*), were unaffected by fructose supplementation. Accordingly, there was no activation of nuclear factor κB (NFκB), exemplified by unchanged expression of IκB and p65 proteins in nuclear extracts (Table 2). Other markers of liver tissue inflammation remained unaltered, mRNA levels of metallothionein-1 and -2 (*mt-1* and *mt-2*) were even reduced, and plasma ALT levels were decreased as well in fructose-supplemented versus control rats, confirming the lack of hepatic inflammation (Table 2). In addition, we did not detect any histological signs of hepatic necrosis or fibrosis (Fig. 3) and mRNA expression of collagenase-1 (*collα1*), a gene involved in liver fibrogenesis, was not significantly altered (Table 2). Similarly, markers of inflammation and fibrosis in vWAT from fructose-supplemented rats were not modified (Table 2).

### Fructose but not glucose supplementation causes an abnormal GTT

We used a second cohort of rats to perform a GTT, where we included an additional group of animals supplemented with a glucose solution adjusted to match the amount of calories provided by fructose (experiment 2). As shown in Table 3, both glucose- and fructose-supplemented groups behaved similarly regarding food and drink consumption: they drank approximately x4-fold more than control rats (p < 0.001), and reduced their solid food consumption x0.6-fold (p < 0.0001). Consequently, the total caloric intake in both groups was almost identical and x1.6-fold higher (p < 0.001) than in control rats. Despite this, body weight and vWAT weight at the end of treatment were not significantly different in the three groups. Plasma triglyceride concentrations were significantly increased (p < 0.01) in sugar-supplemented rats. Plasma insulin levels were also increased (p < 0.05 for fructose-fed rats), whereas glucose concentrations were not changed.

**Figure 4:**
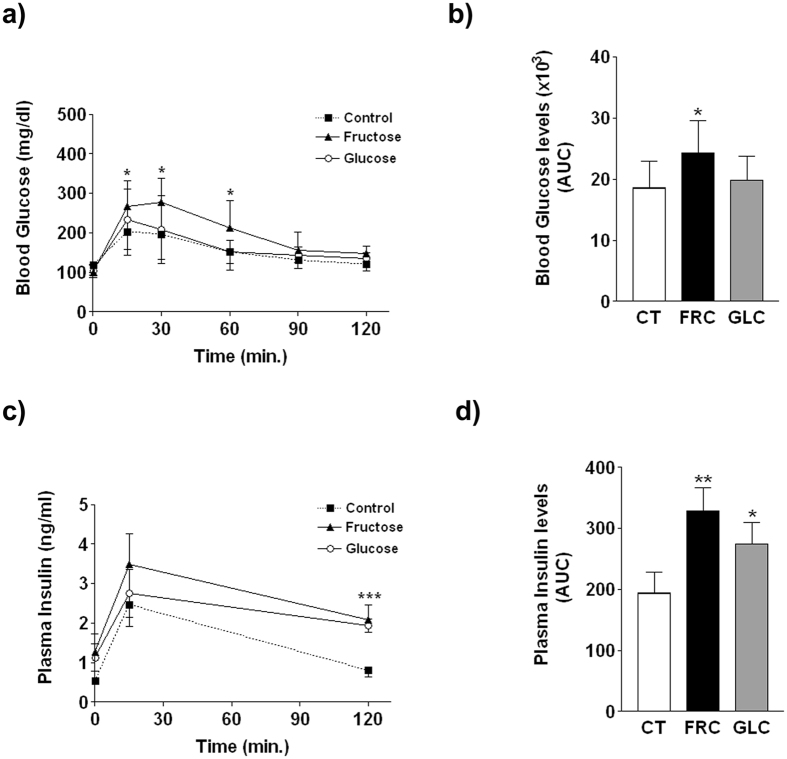
Results of the glucose tolerance test in rats from experiment 2. Blood glucose (**a**) and plasma insulin (**c**) values at different times after intraperitoneal administration of a glucose solution (2 g/kg body weight). Area under the curve (AUC) values for glucose (**b**) and insulin (**d**) concentrations shown in Fig. 1a,c, respectively. Results are the mean ± sd of values from 10 animals/group. *p < 0.05; **p < 0.01; ***p < 0.001 (one-way ANOVA and Bonferroni post test).

The results of the GTT showed that only fructose-supplemented rats displayed increased blood glucose excursions at all time-points after the glucose challenge (Fig. 4a); consequently, the integrated glucose concentration calculated as the area under the curve (AUC) was increased x1.3-fold in fructose- (p < 0.05 vs control) but not in glucose-supplemented rats (Fig. 4b). However, glucose-stimulated insulin levels were significantly increased in both fructose- and glucose-supplemented rats (Fig. 4c,d).

### Increased phosphorylation of forkhead box protein O1 (FoxO1) impairs gluconeogenic gene induction even though insulin-induced Akt phosphorylation is hampered in the liver of fructose-supplemented rats

**Figure 5:**
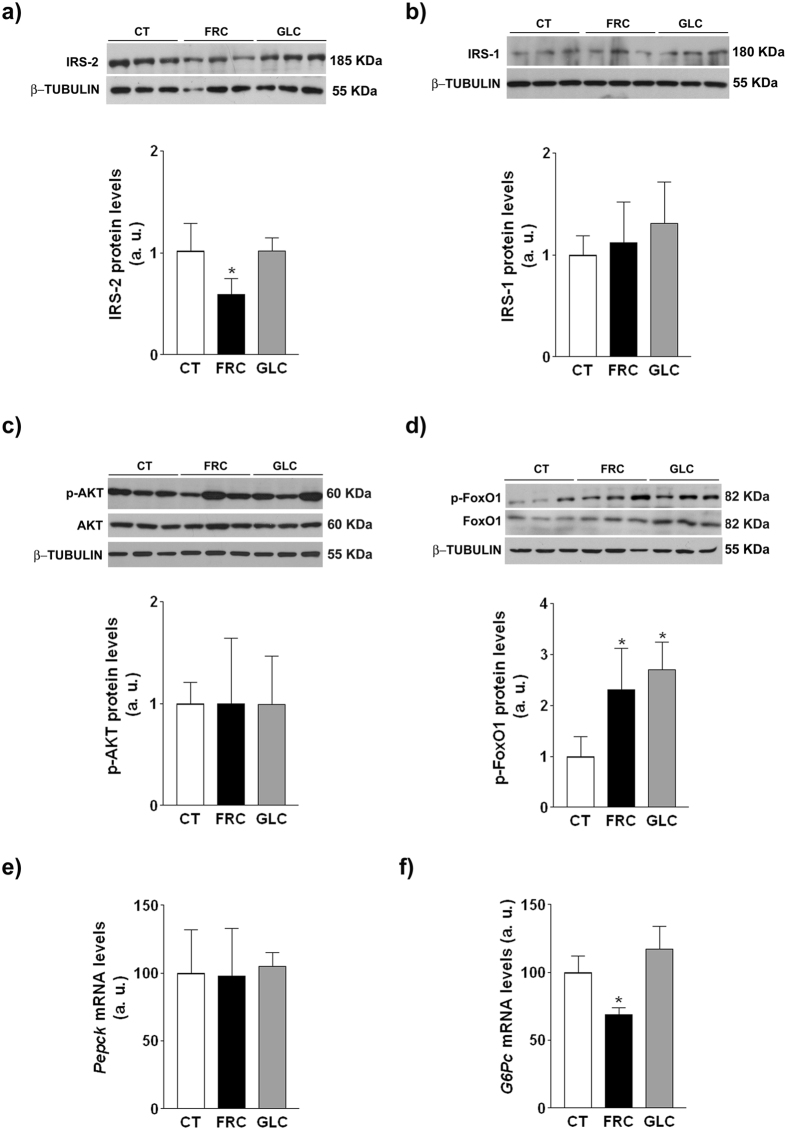
Insulin signaling in hepatic samples. Protein expression of (**a**) IRS-2, (**b**) IRS-1, (**c**) phosphorylated and total AKT, and (**d**) phosphorylated and total FoxO1, and mRNA levels of *pepck* (**e**) and *g6pc* (**f**). Bars represent the mean ± sd of values obtained from n = 4 rats from experiment 2/group. Representative western blots corresponding to 3 different rats for each condition are shown in Fig. a–c. *p < 0.05 (one-way ANOVA and Bonferroni post test).

Fructose-, but not glucose-supplemented rats, exhibited reduced levels of hepatic IRS-2 (x0.59-fold, p < 0.05) (Fig. 5a), whereas IRS-1 expression was not significantly modified by sugar supplementation (Fig. 5b). The abnormal GTT and reduced IRS-2 expression in the liver of fructose-supplemented rats pointed to an impairment in hepatic insulin signaling. Despite the hyperinsulinaemia present in both carbohydrate-supplemented groups (Table 3), phosphorylation of Akt in the liver was not modified (Fig. 5c). However, there was a marked increase in the phosphorylated form of FoxO1 in the livers of fructose- (x2.3-fold, p < 0.05) and glucose-supplemented (x2.7-fold, p < 0.05) rats (Fig. 5d). The expression of major gluconeogenic genes was either unchanged (phosphoenolpyruvate carboxykinase, *pepck;* Fig. 5e) or even reduced (glucose-6-phosphatase, *g6pc*; Fig. 5f).

**Figure 6:**
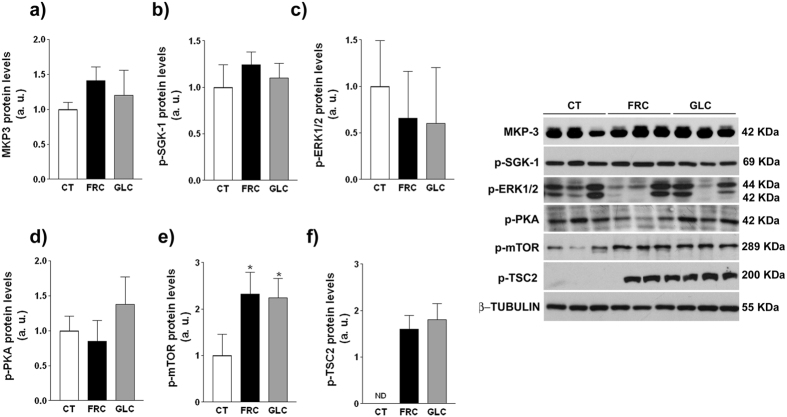
Expression of phosphatases and kinases related to FoxO1 phosphorylation in hepatic samples. Western blot of MKP-3 (**a**), phospho-SGK-1 (**b**), phospho-ERK1/2 (**c**) phospho-PKA (**d**), phospho-mTOR (**e**), and phospho-TSC2 (**f**). Bars represent the mean ± sd of values obtained from n = 4 animals from experiment 2. Representative bands corresponding to 3 different rats per treatment group are shown. ERK: extracellular signal-regulated kinase; MKP-3: MAP kinase phosphatase 3; PKA: protein kinase A; SGK-1: serum- and glucocorticoid- inducible kinase 1. *p < 0.05 (one-way ANOVA and Bonferroni post test).

Possible explanations for increased levels of p-FoxO1 independently from Akt phosphorylation status could include changes in the expression or activity of other kinases or phosphatases, but none of them were significantly affected by carbohydrate supplementation (Fig. 6a–d). Recently, it has been described that FoxO1 phosphorylation is controlled by the mammalian target of rapamycin (mTOR) in rat hypothalamus^25^ and in human smooth muscle cells^26^. As shown in Fig. 6e,f, hepatic samples from both fructose- and glucose-supplemented rats showed an increase in Ser-2481 mTOR and in Thr-1462 tuberous sclerosis complex 2 (TSC2) phosphorylation, indicative of mTOR activation.

**Figure 7:**
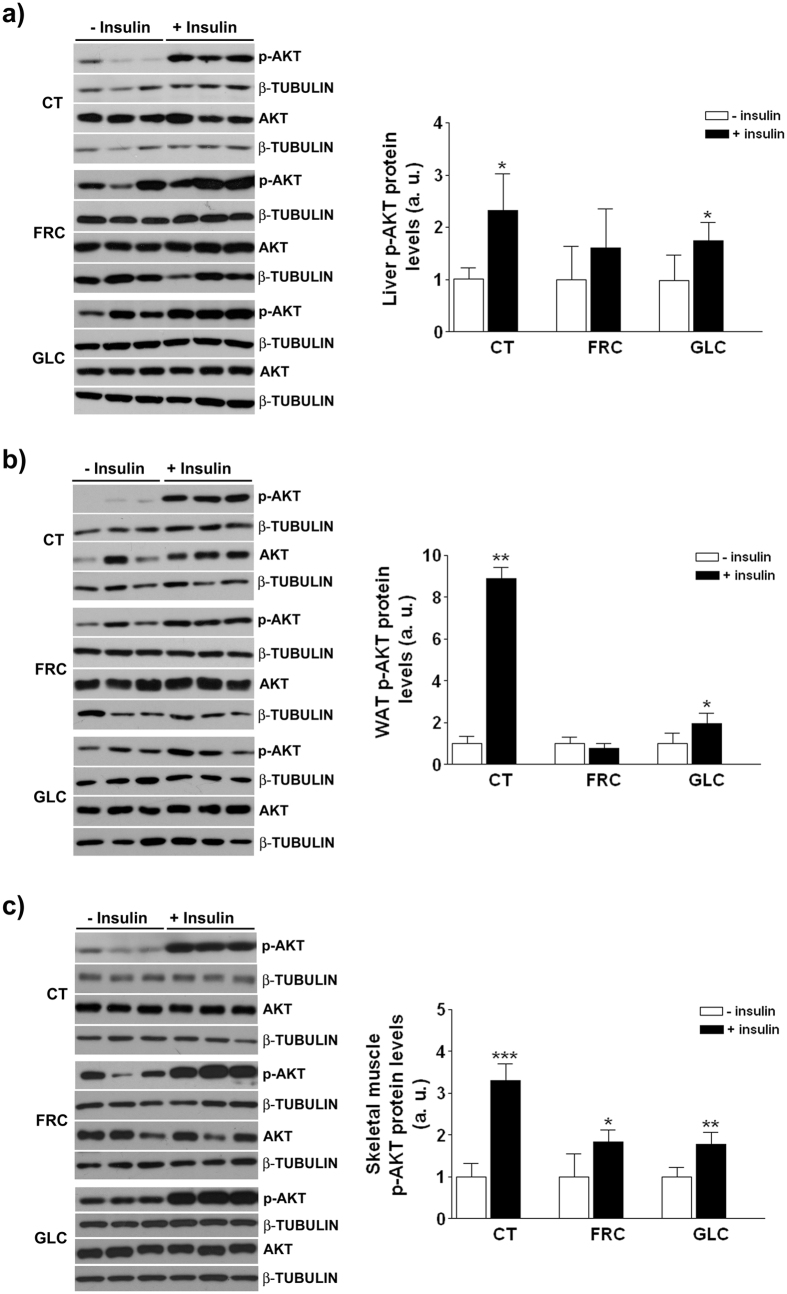
Insulin signaling in target tissues from rats of experiment 2. Protein expression of total and phosphorylated-AKT in the liver (**a**), vWAT (**b**) and skeletal muscle (**c**) of control, glucose- and fructose-supplemented rats injected with saline (n = 4/group) or insulin (n = 6/group). Bars represent the mean ± sd, and representative western blots corresponding to 3 different rats for each condition are shown. *p < 0.05; **p < 0.01; ***p < 0.001 (one-way ANOVA and Bonferroni post test).

To further explore hepatic insulin sensitivity, Akt activation was measured in the liver of control and carbohydrate-supplemented rats 15 minutes after a single injection of saline or insulin (0.15 units/g body weight). Akt phosphorylation was increased x2.3-fold and x1.7-fold (p < 0.05) in the liver of insulin-treated rats from control and glucose groups compared with saline-treated animals, but not in fructose-supplemented rats (Fig. 7a).

### Fructose but not glucose supplementation impairs insulin signaling in vWAT

**Figure 8:**
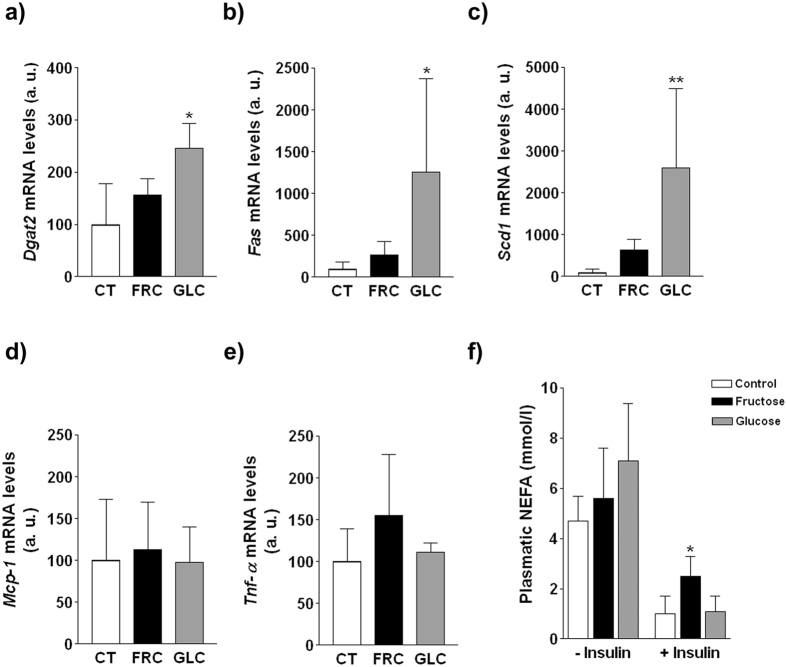
mRNA levels of key enzymes involved in triglyceride synthesis and inflammation in vWAT: (**a**) *dgat*2, (**b**) *fas*, (**c**) *scd1*, (**d**) *mcp-1* and (**e**) *tnfα*. Bars represent the mean ± sd of values obtained from rats from experiment 2 injected with saline (n = 4/group). (**f**) Concentration of NEFA in plasma samples. Bars represent the mean ± sd of values obtained from rats from experiment 2 injected with saline (n = 4/group) or insulin (n = 6/group).*p < 0.05; **p < 0.01 (one-way ANOVA and Bonferroni post test).

Under basal conditions the expression of key enzymes involved in triglyceride synthesis was only significantly increased in vWAT of glucose-supplemented rats (Fig. 8a–c), even though insulin promotes lipogenesis in adipose tissue and both carbohydrate-supplemented groups showed hyperinsulinaemia. On the other hand, sugar supplementation did not promote inflammation in vWAT, as shown by no significant changes in mRNA levels of *mcp-1* and *tnfα* (Fig. 8d,e). When we compared Akt phosphorylation in vWAT after administration of the insulin bolus (Fig. 7b), we observed a striking increase in control rats (x8.9-fold, p < 0.05), a significant but less marked increase in glucose-supplemented rats (x1.9-fold, p < 0.05) and no change in fructose-supplemented rats. As the main effect of insulin on adipose tissue is lipolysis inhibition, we determined plasma NEFA levels after insulin administration. NEFA were reduced in the three experimental groups, although the reduction was of a greater magnitude in control and glucose-supplemented rats (Fig. 8f).

### Fructose, but not glucose supplementation reduces GLUT4 levels in plasma membrane

**Figure 9:**
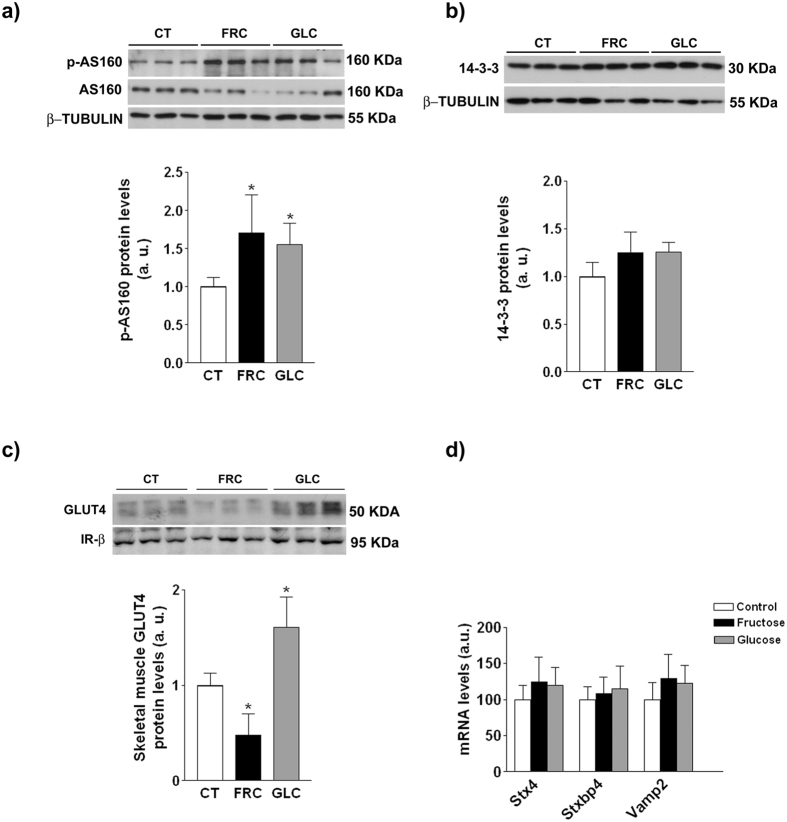
Effects of sugar supplementation on the expression of proteins involved in glucose transport in skeletal muscle. Protein expression of phosphorylated and total AS160 (**a**), 14-3-3 (**b**) in whole skeletal muscle protein extracts, and of GLUT4 in a plasma membrane fraction isolated froms skeletal muscle (**c**) of control, fructose- and glucose-supplemented rats from experiment 2 after insulin injection. Bars represent the mean ± sd of values obtained from n = 6 rats/group. Representative western blots corresponding to 3 different rats for each condition are shown. (**d**) mRNA levels of *vamp2, stx4* and *stxbp4* in skeletal muscle of control, fructose- and glucose-supplemented rats from experiment 2 after insulin injection. Bars represent the mean ± sd of values obtained from n = 6 rats/group. *p < 0.05; **p < 0.01 (one-way ANOVA and Bonferroni post test).

Exogenous insulin administration similarly increased Akt phosphorylation in skeletal muscle from control, glucose- and fructose-supplemented animals (Fig. 7c). AS160 phosphorylation in insulin-treated rats was significantly increased in both fructose and glucose groups (x1.7- and x1.5-fold, respectively, p < 0.05), whereas the total amount of AS160 was not significantly modified (Fig. 9a). The amount of the phosphoprotein-binding protein 14-3-3 was not affected by sugar supplementation (Fig. 9b). However, the expression of GLUT4 in a plasma membrane-enriched fraction was reduced in fructose-supplemented rats and increased in the glucose group (Fig. 9c). To further characterize the process of GLUT4 translocation, we assessed the expression of proteins involved in the fusion of GLUT4 containing vesicles to the plasma membrane, but none of them was significantly modified by sugar supplementation (Fig. 9d).

## Discussion

Here we show that supplementation with 10% w/v liquid fructose for 2 months to female rats causes liver deposition of triglycerides enriched in MUFA with no signs of liver and vWAT inflammation and oxidative stress. Nevertheless, fructose-supplemented rats display an abnormal GTT and an impairment of insulin signaling in the three major insulin-responsive tissues: in vWAT and liver, where fructose inhibits insulin-induced Akt phosphorylation, and in skeletal muscle, where Akt is correctly activated by insulin but GLUT4 expression in the plasma membrane is reduced. These effects are specific to fructose, and not related to the amount of calories ingested, as equicaloric glucose supplementation does not affect tissue-insulin signaling and does not alter the GTT, although at the expense of high plasma insulin levels.

We and others have previously reported fructose-related stimulation of *de novo* lipogenesis and fatty liver in animal models^15^,^27^,^28^,^29^,^30^ and in humans^31^,^32^,^33^. In this complex process, the microsomal enzyme SCD1 is rate-limiting in the hepatic synthesis of palmitoleic and oleic acids, the main species contained in liver lipid droplets. However, there is debate on whether expression of SCD1 causally contributes to the disease or is a mere consequence, i.e., to prevent liver damage by partitioning more lipotoxic intermediate products such as free fatty acids, ceramides and diacylglycerols^34^,^35^. In line with the latter, a recent study in mice overexpressing ChREBP showed that SCD1 induction increases the content of hepatic MUFA while protecting the liver from insulin resistance and hepatic inflammation^13^. Fructose supplementation induces a similar effect in our study: ChREBP is activated (Fig. 1D), SCD1 expression^14^ and activity (Fig. 1C) are increased, and there is a change in the pattern of hepatic triglyceride fatty acids consisting in an increase in MUFA (palmitoleic and oleic) and a decrease in n-6 and n-3 polyunsaturated fatty acids (PUFA) (Fig. 1A), but no inflammation is ensued (Table 2). The last finding is consistent with a trial in healthy humans, reporting that very-high-fructose or glucose intake for 4 weeks does not cause inflammation^36^. The decrease in PUFA could also explain the lack of oxidative stress in fructose-supplemented rats, as PUFA are more prone to oxidation than MUFA, and n-3 PUFA promote lipid oxidation^37^. In addition, inflammation in fructose-supplemented mice has been shown to result from alterations in the intestinal barrier permeability and increased flux of endotoxins to the liver^38^,^39^. We did not find increased plasma endotoxin levels or changes in the expression of related receptors and downstream factors in rats, suggesting that this is a species-specific response of mice to fructose supplementation.

Despite the lack of an inflammatory or oxidative response, we nevertheless observe reduced levels of the main hepatic insulin transducer, IRS-2, in the liver of fructose-supplemented rats, thus suggesting liver insulin resistance. Reduced hepatic IRS-2 was also found in our previous 2-week fructose supplementation study, and we attributed this effect to increased mTOR activity^11^. Besides observing that same increment in mTOR activity within this study, we show an increase in miR-33-3p as a new mechanism contributing to fructose reduction of hepatic IRS2 expression^40^. To our knowledge, this is the first report on the modulation of miRNA-33 by fructose ingestion, although dietary glycaemic load, and specifically diet supplementation with liquid fructose, can alter the miRNA liver expression profile^41^,^42^.

Under equicaloric conditions (see Table 3), we show that fructose but not glucose supplementation induces glucose intolerance. Both glucose- and fructose-supplemented groups responded to the glucose challenge by increasing plasma insulin levels; however, this hyperinsulinaemia only maintained blood glucose concentrations within the normal range in glucose-supplemented animals, whereas glucose levels throughout the GTT were increased by 30% in fructose-supplemented rats (Fig. 4). This difference between the two carbohydrates prompted us to examine whether insulin signaling is also specifically impaired by fructose in target tissues.

In adipose tissue, insulin signals through Akt to reduce lipolysis and NEFA release to the bloodstream^43^,^44^. Our results demonstrate that fructose impairs insulin signaling in vWAT: after administration of an insulin bolus, Akt phosphorylation in vWAT is only blunted in fructose-supplemented rats (Fig. 7b). Crescenzo *et al*. reported reduced p-Akt related to plasma insulin in WAT, and impairment in the capacity of insulin to inhibit lipolysis in cultured adipocytes from male rats fed a fructose-rich solid diet^45^. Although we did not directly measure *in vitro* lipolytic activity, plasma NEFA after insulin administration were more efficiently reduced in control and glucose-fed animals than in the fructose group (Fig. 8f).

In the liver, only fructose supplementation reduces IRS-2 protein expression and insulin-induced Akt phosphorylation (Figs 5a and 7a). Nevertheless, this state of fructose-induced insulin resistance is atypical, as FoxO1 phosphorylation is increased (Fig. 5d) and therefore the expression of gluconeogenic genes is not induced (Fig. 5d,e). It should be noted that FoxO1 phosphorylation is increased in both fructose and glucose groups, suggesting that this effect is independent from Akt and related to the high calorie intake of these animals. Nutrient-rich conditions activate mTOR^46^, and we have previously demonstrated that fructose supplementation activates mTOR through increased phosphorylation^14^. We show here that mTOR is activated to the same extent in both carbohydrate-supplemented groups (Fig. 6e), which is attributable to an almost identical excess in nutrient intake (Table 3). As it has recently been shown that activated mTOR inhibits the activity of FoxO1by increasing its phosphorylation^25^,^26^, we can conclude that the increase in calorie intake in both carbohydrate-supplemented groups leads to mTOR activation, resulting in similar increases in FoxO1 phosphorylation.

One consequence of this atypical hepatic insulin resistance is that the higher glucose levels observed during the GTT in fructose-supplemented rats cannot be explained by an increase in hepatic glucose production. Reduced glucose uptake by skeletal muscle may be an alternative explanation, as this is the main tissue responsible for glucose disposal^47^. Here, we show that after exogenous insulin administration, Akt is phosphorylated to a similar extent in the three experimental groups, but the translocation of the glucose transporter GLUT4 to the plasma membrane is specifically inhibited in fructose-supplemented rats (Fig. 9c). The mechanism for insulin-induced GLUT4 translocation involves the binding of phosphorylated AS160 to 14-3-3, which promotes the movement of GLUT4 storage vesicles to fuse with the plasma membrane^48^. Our results do not allow to establish the molecular mechanism by which GLUT4 translocation to the plasma membrane of skeletal muscle is altered by sugar supplementation, as AS160 phosphorylation is similarly increased in both glucose- and fructose-supplemented rats and neither the amount of 14-3-3 nor the mRNA expression of proteins involved in the fusion of GLUT4 vesicles to the membrane are modified (Fig. 9a,c,d). However, the reduction of GLUT4 expression in the plasma membrane of skeletal muscle suggests that the high plasma glucose levels observed in the GTT in fructose-fed rats are a consequence of reduced glucose uptake in this tissue and not of increased hepatic glucose production. Similar results were observed in male Wistar rats fed a solid diet with 60% fructose for 6 weeks^49^.

Few human intervention studies have determined muscle insulin sensitivity after high fructose consumption, and the most of these did not detect any effect^32^,^50^,^51^,^52^. However, these studies have generally been small, performed on young healthy individuals, and short (1–4 weeks), whereas consumption of fructose-rich diets in the general population may span many years and include people with genetic backgrounds prone to metabolic diseases, such as obesity. In this sense, a longer intervention study (10 weeks) has demonstrated reduced insulin sensitivity in overweight/obese subjects consuming fructose but not glucose at the same % of energy requirements^3^.

We can conclude that with the exception of mTOR activation, molecular changes in liver, vWAT and skeletal muscle are related to fructose-induced insulin resistance, and rather than being directly related to the amount of calories ingested, are produced by fructose itself. The reduction of hepatic IRS-2 in fructose-supplemented rats can be interpreted as the combined effect of mTOR activation (induced by the excess of sugar-related calories consumed) and miRNA induction, an effect that is probably particular to fructose.

One of the strengths of our study is the inclusion of glucose-supplemented rats whose calorie consumption was matched to that of fructose-supplemented animals, allowing us to demonstrate that the effects of fructose on insulin sensitivity are specific and independent from the amount of calories provided by its consumption. Moreover, we want to highlight that in our study we simultaneously assessed the response to insulin and the mechanisms involved in the three target tissues (adipose, liver and skeletal muscle), thus obtaining a complete picture of whole-body insulin sensitivity.

However, our study also presents some limitations, besides the difficulties entailed in directly extrapolating results obtained in experimental animals to humans. These are: 1. We used female rats, and we cannot be sure that the same effects would be observed in male rats; 2. high consumption of sugar-sweetened beverages often cluster with other bad dietary habits in a Western-type diet, high in calories and saturated fats and poor in complex carbohydrates and micronutrients; 3. We used simple sugar solutions with concentrations (10% w/v) very close to those of sugar-sweetened beverages consumed by humans; however, to obtain meaningful results within a reasonable timeframe, rats were given *ad libitum* access to these solutions. As a consequence, around 60% of their total daily energy ingestion came from fructose or glucose, whereas heavy consumers in human populations obtain a maximum of 20–25% of their daily energy intake from simple sugars. In future research projects, we will attempt to address these questions by restricting the amount of total calories obtained from sweetened beverages, while increasing the length of treatments and the type of solid chow consumed.

## Additional Information

**How to cite this article**: Baena, M. *et al*. Fructose, but not glucose, impairs insulin signaling in the three major insulin-sensitive tissues. *Sci. Rep.* **6**, 26149; doi: 10.1038/srep26149 (2016).

## Supplementary Material

Supplementary Information

## Figures and Tables

**Table 1:** Ingested kilocalories, plasma analytes and liver triglycerides of female rats supplemented or not with 10% w/v liquid fructose for 2 months.

	**Control**	**Fructose**
Kcal ingested from solid (2 rats x 2 months)^a^	5604 ± 44	3296 ± 138***
Kcal ingested from líquid (2 rats x 2 months)^a^	0	5825 ± 594
Total ingested kcal (2 rats x 2 months)^a^	5604 ± 44	9121 ± 683***
Plasma insulin (ng/ml)	0.22 ± 0.04	0.42 ± 0.13*
Plasma glucose (mg/dl)	107 ± 14	110 ± 12
Plasma cholesterol (mg/dl)	160 ± 3	164 ± 8
Hepatic triglycerides (mg/mg of protein)	0.011 ± 0.001	0.018 ± 0.005*

AUC: Area Under the Curve. Values are expressed as mean ± SD of 5 controls and 8 fructose-supplemented rats, except for values^a^, which were obtained from 4–6 cages containing 2 rats each. *p < 0.05; ***p < 0.001 (unpaired t test).

**Table 2:** Effects of 10% w/v fructose supplementation for 2 months on markers of oxidative stress, inflammation and fibrosis.

				**Control**	**Fructose**
Oxidative stress	Liver	mRNA	*nrf2*	100 ± 39	55 ± 28*
			*gpx1*	100 ± 3	93 ± 11
			*sod2*	100 ± 10	108 ± 9
Inflammation	Liver	mRNA	*tlr4*	100 ± 14	84 ± 11
			*myd88*	100 ± 7	84 ± 8
			*tnfα*	100 ± 57	115 ± 59
			*nlrp3*	100 ± 55	108 ± 54
			*mcp-1*	100 ± 17	106 ± 19
			*pai-I*	100 ± 10	95 ± 19
			*mt-1*	100 ± 19	40 ± 21***
			*mt-2*	100 ± 40	32 ± 17*
		protein	IκBα	1.00 ± 0.07	0.96 ± 0.06
			p65	1.00 ± 0.09	0.97 ± 0.18
	VWAT	mRNA	*tlr4*	100 ± 51	100 ± 41
			*myd88*	100 ± 49	96 ± 21
			*tnfα*	100 ± 88	92 ± 74
			*mcp-1*	100 ± 38	131 ± 68
	Plasma	Endotoxin (EU/ml)		29.1 ± 28.5	20.1 ± 12.8
		ALT (U/l)		25.7 ± 7.5	18.1 ± 4.2*	
Fibrosis	Liver	mRNA	*collα1*	100 ± 64	119 ± 74	
	vWAT	mRNA	*collα1*	100 ± 51	68 ± 76	

Values are expressed as mean ± SD of 5 controls and 8 fructose-supplemented rats. *Collα1*: collagenase 1, *gpx1*: glutathione peroxidase 1, IκBα: nuclear factor κ-B inhibitor α, *mcp-1*: monocyte chemoattractant protein- 1, *mt*: metallothionein, *myd88*: myeloid differentiation factor 88, *nlrp3*: pyrin domain containing 3 gene, *nrf2*: nuclear factor-E2-related factor-2, *pai-I*: plasminogen activator inhibitor-I, *tlr*: toll-like receptor, *sod2*: superoxide dismutase 2, *tnfα:* tumor necrosis factor α. EU: endotoxin units. U: international units *p < 0.05; ***p < 0.001 (unpaired t test).

**Table 3:** Zoometric parameters and plasma analytes of female rats supplemented or not with 10% w/v liquid fructose or glucose for 2 months.

	**Control**	**Fructose**	**Glucose**
AUC solid food intake (g/[2 rats x 2 months])^a^	1553 ± 79	930 ± 124***	904 ± 100***
AUC liquid intake (ml/[2 rats x 2 months])^a^	2893 ± 711	11448 ± 2713***	12163 ± 701***
Kcal ingested from solid (2 rats x 2 months)^a^	4502 ± 229	2698 ± 359***	2624 ± 289***
Kcal ingested from liquid (2 rats x 2 months)^a^	0	4579 ± 1085	4850 ± 280
Total ingested kcal (2 rats x 2 months)^a^	4502 ± 229	7277 ± 735***	7489 ± 354***
Final body weight (g)	253.2 ± 14.9	261.8 ± 12.3	265.3 ± 16.1
AUC weight (g/[2 rats x 2 months])^a^	13188 ± 721	13479 ± 534	13594 ± 645
vWAT veight (g)	5.6 ± 1.4	6.5 ± 1.7	6.8 ± 1.8
Plasma insulin (ng/ml)	0.54 ± 0.02	1.26 ± 0.46*	1.13 ± ± 0.35
Plasma glucose (mg/dl)	118 ± 25	100 ± 17	104 ± 17
Plasma triglycerides (mg/dl)	128 ± 17	177 ± 40**	159 ± 18**

AUC: Area Under the Curve. Values are expressed as mean ± SD of 10 controls, 10 glucose and 10 fructose-supplemented rats, except for values^a^, which were obtained from 5 cages containing 2 rats each. *p < 0.05; **p < 0.01; ***p < 0.001 (one-way ANOVA and Bonferroni post-test).
