# Supplementary material for: Fructose, but not glucose, impairs insulin signaling in the three major insulin-sensitive tissues
Source: Sci Rep. 2016 May 19;6:26149. doi: 10.1038/srep26149 (PMC4872141; doi:10.1038/srep26149)
Supplement: Supplementary Information [file srep26149-s1.pdf]

Title page

Fructose, but not glucose, impairs insulin signaling in the three major insulin-sensitive tissues

Miguel Baena[1,2,+], Gemma Sangüesa[1,2,+], Alberto Dávalos[3], María-Jesús Latasa[3], Aleix Sala-Vila[4,5], Rosa María Sánchez[1,2,4], Núria Roglans[1,2,4], Juan Carlos Laguna[1,2,4,*] and Marta Alegret[1,2,4,*]

[1]Department of Pharmacology and Therapeutic Chemistry, School of Pharmacy, University of Barcelona, Spain

[2]Institute of Biomedicine, University of Barcelona, Spain

[3]IMDEA Food. CEI UAM+CSIC, Madrid, Spain

[4]CIBER Fisiología de la Obesidad y Nutrición (CIBEROBN), Instituto de Salud Carlos III (ISCIII), Spain

[5]Lipid Clinic, Endocrinology and Nutrition Service, Hospital Clínic, Institut d'Investigacions Biomèdiques August Pi i Sunyer (IDIBAPS), Barcelona, Spain

* Equally contributing senior authors, and authors for correspondence: alegret@ub.edu, jclagunae@ub.edu

[+] Miguel Baena and Gemma Sangüesa contributed equally to this work

Correspondence to: Marta Alegret, Department of Pharmacology and Therapeutic Chemistry, School of Pharmacy, University of Barcelona, Joan XXIII 27-31, Barcelona 08028, Spain; phone: 34 93 4024530 ext 28, fax: 34 93 4035982, e-mail: alegret@ub.edu

Supplemental Table 1: Primers used for RT-PCR

Gen (NCBI Reference)	Primer sequences (forward)	Primer sequences (reverse)	PCR product
***collα1* (NM_001134530.1)**	5'-TTTGTGCTAAACCAGGAGGATATTAA-3'	5'-TGGCTGGATGGGATTTGG-3'	72 bp
***dgat* (NM_001012345.1)**	5'-GGAACCGCAAAGGCTTTGTA-3'	5'-GGAATAGGTGGGAACCAGATCA-3'	65 bp
***fas* (NM_017332.1)**	5'-GGCTCTATGGGTTGCCTAAGC-3'	5'-GGTGGACCCCAAAAAAGGA-3'	78 bp
***g6pc* (NM_013098.2)**	5'-GGCTCACTTTCCCCATCAGG-3'	5'-ATCCAAGTGCGAAACCAAACAG-3'	146 bp
***gpx1* (NM_030826.3)**	5'TCGGTTTCCCGTGCAATC-3'	5'-TGAGGGAATTCAGAATCTCTTCATT-3'	69 bp
***mcp1* (NM_031530.1)**	5'-CTGTCTCAGCCAGATGCAGTTAA-3'	5'-TGGGATCATCTTGCCAGTGA-3'	69 bp
***mt1* (NM_138826.4)**	5'-CTCCTGCACCTGCTCCAGCTC-3'	5'-AGGCACAGCACGTGCACTTGT-3'	152 bp
***mt2* (NM_001137564.1)**	5'-TGTGCCACAGATGGATCCTGC-3'	5'-CAGCAGCTGCACTTGTCCGAA-3'	161 bp
***myd88* (NM_198130.1)**	5'-GCCAGCGAGCTCATTGAGA-3'	5'-TTTGCAGGTAATCGTCAGAAACA-3'	67 bp
***nlrp3* (NM_001191642.1)**	5'-GACGCTACACCCAGCTCCAA-3'	5'-GTCAAGAGTTCATGCTCCCTTTC-3'	70 bp
***nrf2* (XM_006234396.2)**	5'-TGAAGACTGTATGCAGCTTTTGG-3'	5'-GGCAAGCGACTGAAATGTAGGT-3'	85 bp
***pai1* (NM_012620.1)**	5'-TGGCTCAGAACAACAAGTTCAAC-3'	5'-GGCAGTTCCAGGATGTCGTACT-3'	76 bp
***sod2* (NM_017051.2)**	5'-CGTCACCGAGGAGAAGTACCA-3'	5'-AGGCTGAAGAGCAACCTGAGTT-3'	70 bp
***tbp* (NM_001004198.1)**	5'-TGGGATTGTACCACAGCTCCA-3'	5'-CTCATGATGACTGCAGCAAACC-3'	132 bp
***tlr4* (NM_019178.1)**	5'-TTGTTCCTTTCCTGCCTGAGA-3'	5'-TGATCCATGCATTGGTAGGTAATATT-3'	83 bp
***tnfα* (NM_012675.3)**	5'- ATCCGAGATGTGGAACTGGC-3'	5'- CGATCACCCCGAAGTTCAGTA-3'	151bp
***vamp2* (NM_012663.2)**	5'-CAAGTGCAGCCAAGCTCAAG-3'	5'-GCGCAAATCACTCCCAAGAT-3'	76 bp
***stx4* (NM_031125.1)**	5'-GAAGAACGTGGAGCGCATTC-3'	5'-CCAGTTCCTCGTCAGACACC-3'	71 bp
***stxbp4* (NM_001107038.1)**	5'-GAAACAGGAAGCCAAAGCCG-3'	5'-GCCTCTAGCAGACGGATCAC-3'	132 bp
